# The occurrences of consecutive infections after cataract surgeries: random events or a product of mistaken public politics?

**DOI:** 10.6061/clinics/2016(06)01

**Published:** 2016-06

**Authors:** Newton Kara-Junior, Newton Kara José

**Affiliations:** Universidade de São Paulo (USP), Disciplina de Oftalmologia, São Paulo/SP, Brazil

Researchers in the field of clinical epidemiology study the distribution and the determinants of health problems to gain insight into how to address public health programs. Specifically, health professionals focused on the development and monitoring of public health projects should base their actions on clinical efficacy and safety data.

Until the mid-1980s, Brazilian Council of Ophthalmology believed that there was no data concerning cataract blindness in the country and university hospitals offered timely, free surgery to resolve cataract blindness. This concept was demystified when epidemiologic studies were published reporting that individuals indeed became blind due to cataracts 1. In fact, those individuals did not know they had cataracts and most of them did not believe that they could be cured 2. These individuals experienced several barriers to care, such as difficulty in accessing the free-of-charge surgery, a lack of transportation to the hospital and unavailability of family members to take them to their many appointments before surgery 3. Therefore, to reduce those barriers, the Cataract-Free Zone Project was launched. University hospitals were converted into health stations to detect individuals with cataracts and to provide them with all the exams necessary to prepare for surgery on the same day. Surgery was then scheduled at a university hospital after providing the patient with an explanation of the disease and the possibility for recovery 4,5.

In 1998, The National Campaign of Cataract was created by initiative of the Brazilian Council of Ophthalmology (CBO) of the national government (Ministry of Health), with the assistance of medical schools. Access to treatment was made possible by launching Cataract-Free Zone Projects nationwide and the national government established unlimited funding for all surgeries by the Brazilian Public System (SUS). These programs provided the greatest impact on visual health in Brazil and the number of surgeries therefore doubled 6.

Based on epidemiologic studies, all clinical doubts regarding the new public actions were being assuaged and the Cataract Free-Zone Projects were being improved 7. Thus, based on scientific evidence, the ideal lower limit of visual acuity for performing surgery was determined. Details on the disease, treatment and risks to be discussed with the patients and their family members were prepared 8. The efficacy of surgery in restoring the patient’s ability to participate in the labor market has been demonstrated 9; additionally, the financial affordability of performing the surgery using the best available technology was confirmed 10,11. Most importantly, the safety of the broad performance of cataract surgery at university hospitals was confirmed 12.

The surgical capacity of school hospitals has been redesigned to meet the increasing demand. The Ophthalmology Department of the Clinical Hospital of the University of São Paulo, for example, increased the number of residents from 5 to 14 per year, enrolled 20 additional training doctors per year and opened a new surgical center. The number of cataract surgeries performed annually increased from 836 in 1998 to 5,078 in 2005 13.

In 2006, the Ministry of Health discontinued the National Campaign of Cataract due to a lack of funds to pay for all surgeries. Thus, hospitals now have a limited quota for performing surgical procedures. This measure hampered the increasing trend of annually performed surgeries in the country. Consequently, the use of the capacity installed for cataract surgery in public hospitals was reduced and another barrier to the access of the population to the surgery was introduced: a limitation of surgical availability 14.

In spite of all the clinical evidence from published epidemiologic studies confirming the success of the methodology used in the national campaign for the eradication of blindness due to cataracts in Brazil, the national government and several city administrations opted for a new strategy to manage the problem. Presently, the principal public health project for the treatment of cataracts is conducted via public bids, in which private companies, some of which have mobile surgical units, negotiate the lowest price to perform a quota of thousands of surgeries.

This system is different from the previous system, in which public universities bore the responsibility to perform most cataract surgeries and the medical society coordinated the national actions. In the previous system, there was no doubt about the transparency of the results and the quality of the procedures, which were guaranteed by the university ideology and testified by publications of epidemiologic studies. In an invitation to bid system, in which the winner is the company that offers the lowest cost for a procedure, the objective of the contracted company is profit.

As to the current reality, several issues should be addressed. 1) Is the truly needy population being assisted? Doubts have originated from informal reports that some operated patients could be rehabilitated by only replacing their glasses. It was previously shown that the challenge of cataract blindness eradication was the provision of access to the needy, considering that university hospital teams in the former project had to enter particular areas of isolated communities to identify surgical patients; thus, we find it strange that private clinics would find it easy to attract thousands of cases. 2) What is the percentage of surgical complications in those private programs? In general, mobile surgical stations, which are common in north and northeast Brazil, do not offer post-operative follow-up examinations. Most patients who do not experience good progression subsequently visit health care centers in the region. There are no data concerning the clinical safety or the long-term social impact of this kind of private project. 3) How can the quality of the utilized services be evaluated? Making a profit on surgeries through the SUS is not easy. There should be savings in basic services to guarantee income.

Infections in series in some centers do not surprise us. When ethics, quality and transparency, which are common to universities focused on teaching, assistance and research, are neglected in favor of public bids by private companies, with the objective of profit, medical care becomes a form of trade.

There appears to be no argument to justify changing a public health project with confirmed effectiveness, quality and safety to a different strategy with dubious results. Losing the opportunity to prepare new surgeons 15,16 and ignoring the enlargement of the physical structures of public hospitals might hinder the acceptance of future incentive actions for cataract surgery. We believe that substantial transparency is needed regarding the surgical procedures, the individuals receiving these surgeries and the complication rates of these programs. Therefore, cost-effective epidemiologic studies are warranted to provide the society with a justification for the substitution of the university/medical society system for the private sector system, considering the favorable cataract treatment provided by the public health system.
